# Barriers to Cardiovascular and Diabetes Care Among Elderly Rohingya Refugees in Bangladesh: A Qualitative Study of Healthcare Provider Perspectives

**DOI:** 10.3390/healthcare14152310

**Published:** 2026-07-31

**Authors:** Aniqa Keya Begum, Shahaduz Zaman

**Affiliations:** Global Health, Brighton and Sussex Medical School, Brighton BN1 9PX, UK

**Keywords:** Rohingya refugees, non-communicable diseases, healthcare access

## Abstract

Background/Objectives: Non-communicable diseases (NCDs), including cardiovascular disease and diabetes, present an increasing challenge in humanitarian settings. While healthcare services in refugee camps have traditionally prioritised acute and communicable diseases, the long-term management of chronic conditions remains difficult, particularly among older adults. This study explored healthcare providers’ perspectives on barriers to cardiovascular disease and diabetes care among elderly Rohingya refugees living in Cox’s Bazar, Bangladesh. Methods: A qualitative study was conducted using semi-structured interviews with six physicians working in Rohingya refugee camps in Cox’s Bazar, Bangladesh. Participants were recruited through purposive and snowball sampling. Interviews were audio-recorded, transcribed verbatim, and analysed using inductive thematic analysis. Results: Three overarching categories of barriers were identified: service delivery barriers, physical and environmental barriers, and socio-cultural barriers. Healthcare providers described limited diagnostic capacity, restricted medication availability, fragmented continuity of care, transport difficulties, challenging camp terrain, language barriers, low health literacy, and reliance on informal healthcare providers as key challenges affecting long-term management of cardiovascular disease and diabetes. These interconnected barriers were perceived to reduce healthcare access and continuity of care for elderly Rohingya refugees. Conclusions: The findings suggest that humanitarian healthcare systems should strengthen long-term management of non-communicable diseases among ageing refugee populations. Improving diagnostic capacity, ensuring reliable access to essential medicines, enhancing continuity of care, and supporting culturally appropriate health education and outreach services may improve chronic disease management in humanitarian settings. Further research exploring the perspectives of elderly Rohingya refugees is needed to complement healthcare provider perspectives.

## 1. Introduction

Non-communicable diseases (NCDs), including cardiovascular disease and diabetes, are leading causes of morbidity and mortality worldwide and account for a growing burden of disease in low- and middle-income countries [1,2,3]. Effective management of these conditions requires timely diagnosis, continuous access to medication, regular monitoring, and sustained engagement with healthcare services [1,2,3].

The global burden of NCDs among displaced populations has received increasing attention in recent years [4]. Refugees often experience disruption of healthcare services, displacement-related stress, financial insecurity, and barriers to accessing healthcare, all of which may contribute to poor disease control and adverse health outcomes [5,6]. Despite increasing recognition of NCDs as a public health priority in humanitarian contexts, evidence regarding the management of chronic diseases among refugee populations remains limited [7,8,9].

The Rohingya population is one of the largest stateless populations in the world [10]. Following large-scale displacement from Myanmar in 2017, more than 900,000 Rohingya refugees settled in camps in Cox’s Bazar, Bangladesh [11,12]. Healthcare services within the camps are primarily delivered by governmental and non-governmental organisations operating in resource-constrained environments [11]. Although substantial efforts have focused on communicable disease control and emergency healthcare provision, the growing burden of chronic diseases presents additional challenges for healthcare systems [7,11].

Older refugees may face particular difficulties in accessing healthcare. Ageing is associated with increased prevalence of cardiovascular disease, diabetes, reduced mobility, and greater dependence on healthcare services [3]. Older adults living in refugee camps may experience additional barriers related to mobility, transportation, health literacy, social support, and access to healthcare facilities [11,13]. Despite these vulnerabilities, the healthcare needs of older refugees remain underrepresented in the literature [11,13].

Previous studies have identified barriers to healthcare access among refugee populations, including language differences, financial constraints, limited healthcare resources, and difficulties navigating healthcare systems [6,14,15]. However, there is limited research exploring the challenges of managing cardiovascular disease and diabetes among elderly Rohingya refugees in Bangladesh. Understanding these barriers is important for informing healthcare planning and improving the delivery of services for this vulnerable population. Although research examining non-communicable diseases among displaced populations has expanded in recent years, evidence remains concentrated within a limited number of refugee contexts. Older refugees remain particularly underrepresented within the literature despite their increased burden of chronic disease and healthcare needs [9,16]. Understanding healthcare providers’ perspectives on barriers to cardiovascular disease and diabetes care among elderly Rohingya refugees therefore addresses an important evidence gap within humanitarian health research.

This study aimed to explore healthcare providers’ perspectives on the barriers faced by elderly Rohingya refugees in accessing healthcare for cardiovascular disease and diabetes in Cox’s Bazar, Bangladesh.

## 2. Materials and Methods

### 2.1. Study Design

This study employed an exploratory qualitative research design using semi-structured, in-depth interviews to explore healthcare providers’ perspectives on barriers to cardiovascular disease and diabetes care among elderly Rohingya refugees living in Cox’s Bazar, Bangladesh. A qualitative approach was considered appropriate because it enables an in-depth understanding of participants’ experiences, perceptions and interpretations within complex humanitarian settings and facilitates exploration of contextual factors that cannot be adequately captured using quantitative methods [17]. The study aimed to understand healthcare providers’ perspectives on the challenges encountered by elderly Rohingya refugees when accessing healthcare for cardiovascular disease and diabetes, rather than to quantify the prevalence of these barriers.

### 2.2. Study Setting

The study was conducted between 20 and 22 July 2019 within the Rohingya refugee camps in Cox’s Bazar, Bangladesh. At the time of data collection, healthcare services within the camps were delivered through multiple governmental and non-governmental organisations providing primary healthcare, preventive services and referral pathways for secondary care. Participants were recruited through BRAC, a Bangladeshi non-governmental organisation delivering primary healthcare services within the refugee camps. BRAC operates primary healthcare centres that provide essential healthcare services in resource-constrained humanitarian settings [18].

### 2.3. Participants and Recruitment

Participants were recruited using purposive sampling followed by snowball sampling. Initial participants were identified through BRAC because of their direct involvement in providing healthcare to Rohingya refugees [19]. Following each interview, participants were invited to identify other eligible healthcare providers who could contribute relevant perspectives to the study.

Eligible participants were healthcare providers aged over 18 years who had worked within the Rohingya refugee camps for at least three months and had direct clinical experience caring for elderly Rohingya refugees. Six healthcare providers participated in the study, comprising five doctors and one doctor who also held the role of Primary Health Care Centre Manager. All participants were male and worked within BRAC-operated primary healthcare facilities serving the Rohingya refugee camps. Participant characteristics are summarised in Table 1.

Given the focused research question and the relatively homogeneous professional backgrounds of participants, a small sample enabled detailed exploration of healthcare providers’ experiences [20]. Interviews continued until no substantially new concepts relevant to the study objectives were emerging across participants [21]. Six interviews were therefore considered sufficient to provide rich qualitative data addressing the aims of this exploratory study, consistent with recommendations for qualitative interview research.

### 2.4. Data Collection

A semi-structured interview guide was developed following a review of the literature and the study objectives. The interview guide explored healthcare providers’ experiences of managing cardiovascular disease and diabetes among elderly Rohingya refugees, perceived barriers to healthcare access, challenges in service delivery, and potential strategies to improve care.

Face-to-face interviews were conducted in private offices within participants’ workplaces after written informed consent had been obtained. Interviews were conducted between 20 and 22 July 2019, lasted between 16 and 50 min, and were digitally audio-recorded with participants’ permission.

Interviews were conducted primarily in English. An independent translator was available where clarification of occasional Bengali phrases was required. These phrases were subsequently translated into English during transcription to produce complete English-language transcripts. During interviews, the first author made brief contemporaneous notes to aid questioning and record immediate observations. These notes were not used as primary analytical data; analysis was based on the verbatim interview transcripts and audio recordings.

### 2.5. Data Analysis

Audio recordings were transcribed verbatim by the first author. Where Bengali phrases occurred, these were translated into English during transcription with assistance from the translator.

Data were analysed using inductive thematic analysis following the six-phase approach described by Braun and Clarke [22]. The analysis took place in Brighton, United Kingdom. The first author repeatedly read the transcripts to become familiar with the data before manually generating initial codes. Following initial coding, conceptually similar codes were grouped into subthemes, which were subsequently reviewed and refined into three overarching themes through an iterative process informed by repeated engagement with the transcripts and discussion with the supervising author. Participant quotations are presented to illustrate the themes identified while maintaining anonymity through participant identification numbers (P1–P6). A coding framework documenting the development of themes and representative quotations was maintained throughout the analytical process and is available from the corresponding author upon reasonable request.

### 2.6. Researcher Reflexivity and Trustworthiness

The first author conducted all interviews, transcription and primary data analysis. At the time of the study, the first author was undertaking postgraduate study in Global Health and had a prior academic interest in refugee health and non-communicable diseases. To minimise the influence of preconceptions, interviews were conducted using open-ended questions and interpretations were grounded in participants’ verbatim accounts. Although brief contemporaneous notes were made during interviews, formal reflexive field notes were not systematically maintained. Credibility was enhanced through repeated reading of transcripts, discussion of emerging themes with the supervising author, and the use of verbatim quotations to support interpretation.

### 2.7. Ethical Considerations

The study was conducted in accordance with the Declaration of Helsinki and received ethical approval from the Brighton and Sussex Medical School Research Governance and Ethics Committee (Reference: **ER/BSMS7349/1**). Written informed consent was obtained from all participants prior to participation. All interviews were anonymised during transcription, and identifiable information was removed to maintain participant confidentiality.

## 3. Results

Six healthcare providers participated in this study. Participants comprised five doctors and one doctor who also held the role of Primary Health Care Centre Manager. All participants were employed by BRAC and worked within primary healthcare centres serving the Rohingya refugee camps in Cox’s Bazar. Participant characteristics are summarised in Table 1.

Inductive thematic analysis identified three overarching themes relating to barriers to cardiovascular disease and diabetes care among elderly Rohingya refugees from the perspective of healthcare providers: **(1) Physical and Environmental Barriers; (2) Service Delivery Barriers; and (3) Socio-cultural Barriers**. These themes and their associated subthemes are presented in Table 2.

The themes describe healthcare providers’ perceptions of the principal challenges affecting healthcare access and delivery for elderly Rohingya refugees with cardiovascular disease and diabetes. The participants contributing to each subtheme are summarised in Table 2 to provide transparency regarding the development of the thematic analysis.

### 3.1. Physical and Environmental Barriers

Healthcare providers consistently described the physical and environmental characteristics of the refugee camps as major barriers to healthcare access for elderly Rohingya refugees, particularly those with reduced mobility. Difficult terrain, including steep hills and uneven pathways, created challenges for elderly refugees with reduced mobility and limited the ability of ambulances to reach some households.

*“There are some parts which are very raised, hill-top parts and from those parts it is difficult even in normal conditions to move able-bodied persons and to move a disabled person would be even tougher. So even if you want to send an ambulance, you won’t be able to reach them.”*

(P1)

Participants suggested that these geographical challenges were exacerbated during periods of adverse weather, further limiting access to healthcare services for older adults.

Most participants also reported that the remote location of the camps and limited transport options created additional barriers, particularly when patients required referral to facilities outside the camps. Although primary healthcare services were generally located nearby, travel costs posed a challenge for many elderly refugees, especially those with mobility difficulties.

*“For cost of travel, there is definitely an issue because we cannot provide travel assistance to each and every patient… patients who are disabled or have problem moving… have to be taken to us in these motorised vehicles and they are sort of costly.”*

(P1)

Overcrowding and limited space within the camps were also perceived to place pressure on healthcare delivery and infrastructure.

### 3.2. Service Delivery Barriers

Despite the availability of primary healthcare services, participants consistently perceived that limitations in infrastructure, diagnostics and resources reduced the quality and continuity of care for chronic disease management. However, high patient volumes frequently resulted in long waiting times, with acutely unwell patients prioritised through a triage system.

*“Usually, we have a triage protocol […] depending on severity of the disease they are classified, categorised. So, if they’re very ill their wait time is low, but if they are in stable condition, their wait time is long.”*

(P1)

Although healthcare facilities were generally located within reasonable distance of the refugee population, participants highlighted that accessibility remained challenging for elderly refugees with reduced mobility. Health services were largely delivered through walk-in outpatient facilities, with no provision for home-based care. As a result, frail or bedridden elderly individuals often experienced difficulty accessing healthcare services.

*“The elderly people actually can’t walk well. So sometimes they don’t get company, so by how actually, they can’t access to us.”*

(P3)

Participants consistently identified resource limitations as a major barrier to effective management of cardiovascular disease and diabetes. Limited infrastructure, unreliable electricity supplies, and shortages of essential equipment affected both treatment and monitoring. These challenges were particularly evident in the management of diabetes, where insulin storage was difficult because many facilities lacked refrigeration.

*“Storing insulin is nearly impossible and we have to rely on oral anti-diabetics.”*

(P1)

Medication availability was also restricted. Healthcare centres generally provided only a limited range of essential medicines, meaning that some patients required referral elsewhere or were prescribed medications that had to be purchased privately. Most participants reported that this sometimes discouraged patients from seeking care or adhering to treatment.

*“Sometimes we have to prescribe the medicine which is not given by us. They need to buy it from the market. That time, they are hesitate, to go or not.”*

(P4)

Diagnostic capacity within the camps was similarly constrained. Participants described limited access to laboratory investigations, electrocardiography, and diabetes monitoring tests such as HbA1c. As a result, diagnoses were often based on clinical assessment rather than confirmatory investigations, making long-term monitoring more difficult.

*“No it’s not available yet. That’s why we cannot do better follow up and prognosis for them. We have to rely solely on RBS and FBS.”*

(P1)

Several participants also highlighted a lack of specialist expertise and advanced diagnostic services for cardiovascular disease management.

*“We don’t have any instrumental facility that we diagnose or confirm. We have a probable diagnosis. We can’t confirm.”*

(P4)

Some participants also identified continuity of care as a significant challenge in managing chronic disease. Refugees were able to access services from multiple organisations, and the absence of an integrated medical record system limited communication between providers. This often resulted in repeated assessments and fragmented care.

*“They are going to another facility and are getting the treatment from the very beginning again. So it breaks the continuity of the services.”*

(P6)

Finally, participants perceived that geriatric care and chronic disease management received less attention than acute illnesses within the humanitarian response. Several felt that services remained primarily focused on communicable diseases and emergency care rather than the long-term management needs of older adults living with chronic conditions.

*“Since beginning, we are trying to manage acute illness, like respiratory infection. Now we try to concentrate on chronic disease and geriatric care, but I think there is no available service for geriatric people.”*

(P2)

### 3.3. Socio-Cultural Barriers

Healthcare providers described several socio-cultural factors that influenced healthcare access and long-term management of cardiovascular disease and diabetes among elderly Rohingya refugees. Key barriers included language differences, limited health literacy, cultural beliefs surrounding illness and ageing, and healthcare-seeking behaviours.

Language barriers were consistently described as a major obstacle to communication between healthcare providers and refugee patients. All participants reported difficulties explaining diagnoses, treatment plans, and health information, particularly for chronic diseases requiring long-term management. Although interpreters and Rohingya community staff were used to facilitate communication, translation was not always accurate and sometimes resulted in misunderstanding or confusion.

*“The language is a big barrier at every point, to counsel them, or to convince them at the level of our attention. What we want to say, what we want to provide services to them.”*

(P6)

*“Usually they are confused as to what is being told to them. Because of the language.”*

(P6)

Participants believed these communication barriers affected patients’ understanding of chronic disease, treatment plans and long-term medication adherence.

Participants also highlighted limited health literacy as an important barrier. Many elderly refugees were perceived to have little understanding of cardiovascular disease or diabetes, with some remaining unaware that they had these conditions. Participants reported that diagnoses were often incidental findings and that explaining chronic disease processes to patients could be challenging because of low educational attainment.

*“Most of them do not know what diabetes is. Their education level is very low and to give them an idea of how a disease may happen, it takes a lot of effort.”*

(P1)

Most participants believed that limited awareness also influenced lifestyle behaviours. Smoking, tobacco use, and high dietary salt intake were described as common risk factors within the refugee population. Conversely, participants recognised that many refugees maintained physically active lifestyles, which they perceived as potentially protective against chronic disease.

*“Rohingya people very much like dry fish. They’re taking more salt I think, that’s because there is more cardiovascular patient prevailing in the community.”*

(P2)

Healthcare-seeking behaviours were also influenced by cultural beliefs and trust in familiar community members. Participants reported that some refugees preferred traditional healers, spiritual remedies, or informal pharmacists rather than healthcare professionals. Familiarity and trust within the community were often perceived as more influential than professional qualifications.

*“They tend to provide more trust to people who they know, even if it costs money. They trust that reputation more than our certification.”*

(P1)

Participants suggested that many refugees sought healthcare only after symptoms became severe, resulting in delayed presentation and treatment.

*“They have tendency of taking, seeking help in very late situations.”*

(P1)

Gender norms were described as an additional barrier, particularly for older women. Most participants reported that some female refugees were initially reluctant to discuss health concerns with male healthcare providers or attend healthcare facilities independently. However, several participants felt that these barriers had improved over time through community engagement and health education initiatives.

*“At first any female patient could not give history to a male doctor, but nowadays they are almost free with us and they don’t feel shy to speak or talk to male doctor.”*

(P4)

Participants further reported misconceptions regarding chronic disease treatment and medication adherence. Some refugees were perceived to believe that chronic diseases could be cured after a single treatment episode and therefore struggled to understand the need for lifelong medication. Fear of future treatment costs, based on previous experiences in Myanmar, was also reported to affect adherence.

*“They have a tendency of thinking that all diseases can be treated in one visit. That a disease might need lifelong treatment, this perception is not traditional to them.”*

(P1)

Participants further suggested that cultural perceptions of ageing influenced the priority given to healthcare for older adults. Some believed that elderly individuals were viewed as requiring less medical intervention, particularly in the context of religious and end-of-life beliefs.

*“They do not think that elderly people need a whole lot of medication to keep sustaining their life. Simply put, elderly medication is not a priority for them.”*

(P1)

## 4. Discussion

This study explored healthcare providers’ perspectives on barriers to cardiovascular disease and diabetes care among elderly Rohingya refugees in Cox’s Bazar, Bangladesh. The findings identified three broad categories of barriers: physical and environmental barriers, service delivery barriers, and socio-cultural barriers. Collectively, these findings suggest a mismatch between humanitarian healthcare delivery models, which have historically focused on acute and communicable diseases, and the long-term management needs of ageing refugee populations living with chronic diseases. The interaction between service delivery limitations, environmental constraints, and socio-cultural factors creates substantial challenges for elderly refugees requiring ongoing care for cardiovascular disease and diabetes, highlighting the need for humanitarian health systems to evolve beyond an acute-care model to better address the long-term needs of ageing refugee populations.

### 4.1. Service Delivery Barriers

Participants consistently identified limitations in healthcare infrastructure, diagnostic capacity, medication availability, and continuity of care as major barriers to chronic disease management. These findings are consistent with previous research demonstrating that non-communicable disease services are often underdeveloped within humanitarian settings, where resources are typically prioritised towards acute and infectious diseases [23,24].

A particularly important finding was the limited availability of diagnostic investigations and disease monitoring tools. Participants described difficulties accessing investigations such as electrocardiography and HbA1c testing, limiting both diagnosis and long-term monitoring of chronic disease. These observations are consistent with previous research among refugee populations in Palestine and other humanitarian settings, where inadequate diagnostic capacity has been associated with poorer disease control and delayed treatment [23,24]. These findings highlight the importance of strengthening the diagnostic infrastructure as part of efforts to improve chronic disease management in refugee settings.

Medication availability also emerged as a significant challenge. Participants reported restricted access to a range of cardiovascular medications and difficulties storing insulin because of unreliable electricity supplies and limited refrigeration capacity. Effective management of cardiovascular disease and diabetes requires consistent access to medication and monitoring, and interruptions in treatment may contribute to disease progression and avoidable complications [25]. Together, these findings reinforce the need for humanitarian health systems to move beyond episodic acute care towards integrated models capable of supporting long-term management of non-communicable diseases.

Participants also highlighted the lack of specialist expertise and integrated systems of care. The absence of electronic medical records and the ability of patients to access multiple providers created challenges for continuity of care. Similar concerns have been identified in other refugee settings, where fragmented healthcare delivery can lead to duplication of services, inconsistent treatment, and reduced treatment adherence [25,26]. Given the chronic nature of cardiovascular disease and diabetes, continuity of care is essential for achieving favourable health outcomes. Without mechanisms to ensure continuity of care, chronic disease management becomes fragmented, increasing the risk of poor disease control and avoidable complications among elderly refugee populations. Although healthcare services within the camps were provided free of charge, participants suggested that concerns regarding future treatment costs remained a barrier to healthcare utilisation. Previous studies have similarly demonstrated that financial uncertainty can influence healthcare-seeking behaviour, even where services are subsidised or provided free at the point of access [27]. This finding highlights the importance of addressing both actual and perceived financial barriers when designing chronic disease services.

### 4.2. Socio-Cultural Barriers

The findings demonstrate that socio-cultural factors play an important role in shaping access to healthcare among elderly Rohingya refugees. Language barriers were consistently identified as a major challenge, affecting communication between healthcare providers and patients and limiting the effective delivery of health information. Comparable challenges have been reported across refugee populations globally, where language differences have been associated with poorer understanding of disease, reduced adherence to treatment, and lower engagement with healthcare services [15,28,29,30]. Although interpreters and Rohingya community workers were used within the camps, participants reported that communication difficulties remained common.

Limited health literacy also emerged as a significant barrier. Healthcare providers consistently described low levels of understanding regarding cardiovascular disease, diabetes, and the need for long-term treatment. This may contribute to delayed presentation, poor treatment adherence, and difficulties engaging with preventative health measures. Similar associations between health literacy and health outcomes have been reported among refugee populations and other socially marginalised groups [31,32,33]. Participants further highlighted the prevalence of behavioural risk factors, including tobacco use and high dietary salt intake, suggesting that culturally appropriate health education interventions may be beneficial.

Trust and healthcare-seeking behaviours were also important influences on access to care. Participants reported that some refugees preferred traditional healers, spiritual remedies, or informal pharmacists rather than healthcare professionals. This aligns with evidence from other refugee and migrant populations, where trust in familiar community members may outweigh trust in formal healthcare systems [34]. Delayed presentation to healthcare services was frequently attributed to these beliefs, as well as limited awareness of chronic disease symptoms.

Gender norms represented an additional barrier to healthcare access, particularly for older women. Participants described reluctance among some female refugees to consult male healthcare providers, although they also reported improvements over time through community engagement and awareness programmes. Similar gender-related barriers have been identified among refugee populations in other settings [35]. These findings highlight the importance of culturally sensitive healthcare delivery models that recognise the influence of social and gender norms on healthcare utilisation.

A novel finding of this study was the influence of beliefs surrounding ageing and the end of life. Participants suggested that some elderly refugees perceived medical treatment as less important in later life, particularly when viewed through religious or cultural understandings of mortality. These beliefs may contribute to reduced prioritisation of chronic disease management and lower treatment adherence. Further research exploring elderly refugees’ own perspectives on ageing, chronic disease, and healthcare utilisation would be valuable.

### 4.3. Physical and Environmental Barriers

Healthcare providers consistently described physical and environmental characteristics of the refugee camps as significant barriers to healthcare access. Difficult terrain, overcrowding, limited transportation, and the remote location of the camps were all perceived to reduce access to healthcare services, particularly for elderly refugees with mobility limitations. These environmental barriers frequently interacted with service delivery limitations, further restricting access to chronic disease care among older adults. These findings are consistent with previous studies demonstrating that physical accessibility remains an important determinant of healthcare utilisation among displaced populations and individuals living with disabilities [36,37,38].

The absence of home-based healthcare services was identified as a particular challenge for frail and bedridden patients. Even where healthcare facilities were geographically close, physical barriers could prevent elderly refugees from attending appointments. These findings suggest that healthcare accessibility should be considered not only in terms of service availability but also in relation to the ability of vulnerable populations to physically reach and utilise those services. Outreach services, community-based care models, and targeted support for individuals with mobility limitations may help reduce these barriers [39].

### 4.4. Strengths and Limitations

This study provides insight into barriers affecting cardiovascular disease and diabetes care among elderly Rohingya refugees from the perspective of healthcare providers working directly within the refugee camps. The qualitative design allowed for an in-depth exploration of healthcare delivery challenges within a complex humanitarian setting. To our knowledge, few qualitative studies have specifically explored healthcare providers’ perspectives on barriers to cardiovascular disease and diabetes care among elderly Rohingya refugees, providing novel insight into chronic disease management within a protracted humanitarian setting.

Several limitations should be acknowledged. The study included a small sample of six healthcare providers from a single organisation, which may limit the transferability of findings to other humanitarian contexts. In addition, only healthcare providers were interviewed; the perspectives of elderly Rohingya refugees themselves were not explored. Consequently, some barriers identified by providers may differ from those perceived by patients. Future studies incorporating refugee perspectives would provide a more comprehensive understanding of healthcare access challenges.

Furthermore, the interviews were conducted in 2019. The interval between data collection and publication reflects the first author’s completion of undergraduate medical training, subsequent postgraduate clinical training, and redevelopment of the original dissertation into a manuscript suitable for publication. Although healthcare provision within the camps has evolved over time, many of the structural barriers identified remain consistent with the more recent literature on chronic disease management in humanitarian settings. The findings should therefore be interpreted within the context of the study period.

### 4.5. Implications for Practice and Future Research

The findings highlight the need for humanitarian healthcare systems to adapt to the growing burden of non-communicable diseases among ageing refugee populations. While humanitarian responses have traditionally prioritised communicable diseases and acute healthcare needs, the findings of this study suggest that greater attention should be given to strengthening long-term chronic disease management within refugee settings. Improving diagnostic capacity, ensuring reliable access to essential medicines, enhancing continuity of care through integrated medical records, and expanding culturally appropriate health education programmes may improve the management of cardiovascular disease and diabetes. Consideration should also be given to outreach and home-based healthcare services for older adults with reduced mobility, together with workforce development and investment in primary healthcare infrastructure capable of supporting long-term management of non-communicable diseases.

Future research should explore the perspectives of elderly Rohingya refugees themselves, evaluate interventions designed to improve chronic disease care in humanitarian settings, and examine how humanitarian health systems can better respond to the growing burden of non-communicable diseases among ageing displaced populations.

## 5. Conclusions

This study explored healthcare providers’ perspectives on barriers to cardiovascular disease and diabetes care among elderly Rohingya refugees in Cox’s Bazar, Bangladesh. The findings identified multiple interconnected barriers, including limitations in healthcare infrastructure and resources, restricted diagnostic and treatment capacity, physical barriers to accessing services, and socio-cultural challenges such as language differences, low health literacy, and healthcare-seeking behaviours.

The findings highlight a mismatch between humanitarian healthcare systems, which have traditionally focused on acute and communicable diseases, and the long-term management needs of ageing refugee populations living with chronic disease. Addressing these challenges will require strengthening diagnostic capacity, improving medication availability, enhancing continuity of care, and developing culturally appropriate health education strategies. Consideration should also be given to outreach and home-based services for elderly refugees with limited mobility.

As the burden of non-communicable diseases continues to rise within displaced populations, greater attention must be given to integrating chronic disease management into humanitarian healthcare responses to ensure that older refugees are not left behind.

## Figures and Tables

**Table 1:** Characteristics of study participants.

Participant	Professional Role	Gender	Organisation	Healthcare Setting
**P1**	Doctor	Male	BRAC	Primary Healthcare Centre
**P2**	Doctor/Primary Healthcare Centre Manager	Male	BRAC	Primary Healthcare Centre
**P3**	Doctor	Male	BRAC	Primary Healthcare Centre
**P4**	Doctor	Male	BRAC	Primary Healthcare Centre
**P5**	Doctor	Male	BRAC	Primary Healthcare Centre
**P6**	Doctor	Male	BRAC	Primary Healthcare Centre

**Table 2:** Themes and subthemes identified during thematic analysis.

Main Theme	Subthemes	Participants Contributing to Subtheme
Physical and Environmental Barriers	Difficult terrain and environmental conditions	P1, P3
	Remote location of healthcare facilities	P4
	Limited transport and ambulance access	P1, P2, P3, P4, P5
	Limited access for frail and bedridden patients	P1, P3
	Overcrowding and long waiting times	P1, P2, P4, P5, P6
	Lack of home-based care	P2, P6
Service Delivery Barriers	Availability of healthcare services within camps	P2, P4, P5, P6
	Limited diagnostic investigations	P1, P2, P3, P4, P5, P6
	Limited medication availability and storage	P1, P2, P3, P4, P5, P6
	Referral pathways	P1, P3, P4, P5, P6
	Lack of specialist expertise	P2, P4
	Coordination and continuity of care	P1, P2, P4, P6
	Financial concerns regarding treatment	P1
Socio-cultural Barriers	Language barriers	P1, P2, P3, P4, P5, P6
	Health literacy and disease knowledge	P1, P2, P3, P4, P6
	Trust and healthcare-seeking behaviours	P1, P6
	Lifestyle factors and behavioural risks	P2, P3, P4, P6
	Perceptions of ageing and end of life	P1, P6
	Gender norms	P4, P6

## Data Availability

The datasets generated and analysed during the current study are not publicly available due to the sensitive nature of the qualitative interview data and the potential risk of participant identification. De-identified data supporting the findings of this study are available from the corresponding author upon reasonable request and subject to ethical approval and data protection requirements.

## References

[1] Daar A.S., Singer P.A., Persad D.L., Pramming S.K., Matthews D.R., Beaglehole R., Bernstein A., Borysiewicz L.K., Colagiuri S., Ganguly N. (2007). Grand challenges in chronic non-communicable diseases. Nature.

[2] Boutayeb A., Boutayeb S. (2005). The burden of non-communicable diseases in developing countries. Int. J. Equity Health.

[3] World Health Organisation (2016). Global Report on Diabetes.

[4] Spiegel P.B., Checchi F., Colombo S., Paik E. (2010). Health-care needs of people affected by conflict: Future trends and changing frameworks. Lancet.

[5] Doocy S., Lyles E., Akhu-Zaheya L., Burton A., Burnham G. (2016). Health service access and utilization among Syrian refugees in Jordan. Int. J. Equity Health.

[6] Langlois E.V., Haines A., Tomson G., Ghaffar A. (2016). Refugees: Towards better access to health-care services. Lancet.

[7] Cetorelli V., Burnham G., Shabila N. (2017). Health needs and care seeking behaviours of Yazidis and other minority groups displaced by ISIS into the Kurdistan Region of Iraq. PLoS ONE.

[8] Ruby A., Knight A., Perel P., Blanchet K., Roberts B. (2015). The effectiveness of interventions for non-communicable diseases in humanitarian crises: A systematic review. PLoS ONE.

[9] Saqib S.M., Ziaei S., Puthoopparambil S.J. (2025). Differences in healthcare access between Rohingya refugees and their host community in Cox’s Bazar, Bangladesh. Front. Public Health.

[10] Milton A.H., Rahman M., Hussain S., Jindal C., Choudhury S., Akter S., Ferdousi S., Mouly T.A., Hall J., Efird J.T. (2017). Trapped in statelessness: Rohingya refugees in Bangladesh. Int. J. Environ. Res. Public Health.

[11] Inter Sector Coordination Group (2018). Situation Report: Rohingya Refugee Crisis, Cox’s Bazar, 5 July 2018.

[12] Bhatia A., Mahmud A., Fuller A., Shin R., Rahman A., Shatil T., Sultana M., Morshed K.A.M., Leaning J., Balsari S. (2018). The Rohingya in Cox’s Bazar: When the stateless seek refuge. Health Hum. Rights.

[13] Hatzidimitriadou E. (2010). Migration and ageing: Settlement experiences and emerging care needs of older refugees in developed countries. Hell. J. Psychol..

[14] Bozorgmehr K., Razum O. (2015). Effect of restricting access to health care on health expenditures among asylum-seekers and refugees: A quasi-experimental study in Germany, 1994–2013. PLoS ONE.

[15] Chuah F.L.H., Tan S.T., Yeo J., Legido-Quigley H. (2018). The health needs and access barriers among refugees and asylum-seekers in Malaysia: A qualitative study. Int. J. Equity Health.

[16] Akik C., Kiapi L., Sibai A.M., Njagi S., Zaitouny N., Fouad F., Mayoufi M., Tessema M.T. (2025). Research priorities for cardiometabolic syndrome in humanitarian settings: A global consensus-based agenda. J. Migr. Health.

[17] Aspers P., Corte U. (2019). What is qualitative in qualitative research. Qual. Sociol..

[18] Nasar K.S., Akhter I., Khaled N., Hossain M.R., Majid T., Tamanna S., Rahman A., Ahmed R., Hasan M.T., Quayyum Z. (2019). Health Care Needs and Health Seeking Behaviour Among Rohingya Refugees in Cox’s Bazar: Study in Selected Camps Served by BRAC’s Health Care Facilities.

[19] Mammen S., Sano Y. (2012). Gaining access to economically marginalized rural populations: Lessons learned from nonprobability sampling. Rural Sociol..

[20] Crouch M., McKenzie H. (2006). The logic of small samples in interview-based qualitative research. Soc. Sci. Inf..

[21] Guest G., Bunce A., Johnson L. (2006). How many interviews are enough? An experiment with data saturation and variability. Field Methods.

[22] Braun V., Clarke V. (2006). Using thematic analysis in psychology. Qual. Res. Psychol..

[23] Shahin Y., Kapur A., Seita A. (2015). Diabetes care in refugee camps: The experience of UNRWA. Diabetes Res. Clin. Pract..

[24] Wali N., Renzaho A.M., Rillotta F., McNamara B., Metta E., Puvimanasinghe T. (2018). Integrating human rights approaches into public health practices and policies to address health needs amongst Rohingya refugees in Bangladesh: A systematic review and meta-ethnographic analysis. Arch. Public Health.

[25] Gammouh O.S., Al-Smadi A.M., Tawalbeh L.I., Khoury L.S. (2015). Chronic diseases, lack of medications, and depression among Syrian refugees in Jordan, 2013–2014. Prev. Chronic Dis..

[26] Amara A.H., Aljunid S.M. (2014). Noncommunicable diseases among urban refugees and asylum-seekers in developing countries: A neglected health care need. Glob. Health.

[27] Boise L., Tuepker A., Gipson T., Vigmenon Y., Soule I., Onadeko S. (2013). African refugee and immigrant health needs: Report from a community-based house meeting project. Prog. Community Health Partnersh..

[28] Davitian K., Noack P., Eckstein K., Hübner J., Ahmadi E. (2024). Barriers of Ukrainian refugees and migrants in accessing German healthcare. BMC Health Serv. Res..

[29] Moezzi S.M.I., Etemadi M., Lankarani K.B., Behzadifar M., Katebzada H., Shahabi S. (2024). Barriers and facilitators to primary healthcare utilization among immigrants and refugees of low and middle-income countries: A scoping review. Glob. Health.

[30] Pitta A., Tzitiridou-Chatzopoulou M., Tsiotsias A., Savvidis S. (2026). Bridging the Language Gap in Healthcare: A Narrative Review of Interpretation Services and Access to Care for Immigrants and Refugees in Greece and Europe. Healthcare.

[31] Kesserawi S., Hübner J., Ahmadi E. (2026). Health literacy, Trust in doctors, Acculturation—Analysis of patient satisfaction of Arab immigrants in general medical care in Germany. BMC Health Serv. Res..

[32] Riggs E., Yelland J., Duell-Piening P., Brown S.J. (2016). Improving health literacy in refugee populations. Med. J. Aust..

[33] Wångdahl J., Lytsy P., Mårtensson L., Westerling R. (2018). Poor health and refraining from seeking healthcare are associated with comprehensive health literacy among refugees: A Swedish cross-sectional study. Int. J. Public Health.

[34] Chiarenza A., Dauvrin M., Chiesa V., Baatout S., Verrept H. (2019). Supporting access to healthcare for refugees and migrants in European countries under particular migratory pressure. BMC Health Serv. Res..

[35] Floyd A., Sakellariou D. (2017). Healthcare Access for Refugee Women with Limited Literacy: Layers of Disadvantage. Int. J. Equity Health.

[36] Elder B.C. (2015). Stories from the margins: Refugees with disabilities rebuilding lives. Soc. Without Bord..

[37] European Union Agency for Fundamental Rights (2016). Thematic Focus: Migrants with Disabilities.

[38] Humanity & Inclusion (2015). Disability in Humanitarian Contexts: Views from Affected People and Field Organisations.

[39] Reilly R. (2010). Disabilities among refugees and conflict-affected populations. Forced Migr. Rev..

